# Convergent degeneration of olfactory receptor gene repertoires in marine mammals

**DOI:** 10.1186/s12864-019-6290-0

**Published:** 2019-12-12

**Authors:** Ake Liu, Funan He, Libing Shen, Ruixiang Liu, Zhijun Wang, Jingqi Zhou

**Affiliations:** 1 Faculty of Biological Science and Technology, Changzhi University, Changzhi, Shanxi 046011 People’s Republic of China; 2 School of Life Sciences, Fudan University, Shanghai, 200438 People’s Republic of China; 3 Institute of Neuroscience, Shanghai Institute for Biological Sciences, Chinese Academy of Sciences, Shanghai, 200031 People’s Republic of China; 4 Department of Chemistry, Changzhi University, Changzhi, Shanxi 046011 People’s Republic of China; 5 School of Public Health, Shanghai Jiao Tong University School of Medicine, Shanghai, 200025 People’s Republic of China

**Keywords:** Olfactory receptors, Marine mammals, Convergent degeneration, Gene gain, Gene loss, Orthologous gene groups

## Abstract

**Background:**

Olfactory receptors (ORs) can bind odor molecules and play a crucial role in odor sensation. Due to the frequent gains and losses of genes during evolution, the number of OR members varies greatly among different species. However, whether the extent of gene gains/losses varies between marine mammals and related terrestrial mammals has not been clarified, and the factors that might underlie these variations are unknown.

**Results:**

To address these questions, we identified more than 10,000 members of the OR family in 23 mammals and classified them into 830 orthologous gene groups (OGGs) and 281 singletons. Significant differences occurred in the number of OR repertoires and OGGs among different species. We found that all marine mammals had fewer OR genes than their related terrestrial lineages, with the fewest OR genes found in cetaceans, which may be closely related to olfactory degradation. ORs with more gene duplications or loss events tended to be under weaker purifying selection. The average gain and loss rates of OR genes in terrestrial mammals were higher than those of mammalian gene families, while the average gain and loss rates of OR genes in marine mammals were significantly lower and much higher than those of mammalian gene families, respectively. Additionally, we failed to detect any one-to-one orthologous genes in the focal species, suggesting that OR genes are not well conserved among marine mammals.

**Conclusions:**

Marine mammals have experienced large numbers of OR gene losses compared with their related terrestrial lineages, which may result from the frequent birth-and-death evolution under varied functional constrains. Due to their independent degeneration, OR genes present in each lineage are not well conserved among marine mammals. Our study provides a basis for future research on the olfactory receptor function in mammals from the perspective of evolutionary trajectories.

## Background

Olfaction plays an important role in the survival of most mammals, thus helping mammals detect food, avoid danger, and identify mates, offspring, and territory [1–3]. Olfactory receptors (ORs) can bind odor molecules and are crucial in olfactory sensation [1, 2]. Buck and Axel first identified the OR gene in rats in 1991 and won the 2004 Nobel Prize for their achievement [1]. These receptors are widely distributed in animals, including terrestrial vertebrates, fish, arthropods and other animals. Over 1000 genes have been found in the olfactory gene family, which is the largest gene family known thus far [1]. In vertebrates, including humans, ORs are located on the olfactory receptor cells, which are abundant and concentrated in a small area behind the nasal cavity and are formed from olfactory epithelial tissue.

Each OR is a G protein-coupled receptor (GPCR) that has seven alpha helix transmembrane domains, which together constitute a region of approximately 310 amino acid residues. There is no intron insertion in the coding region of OR genes, and introns are usually located in the 5’UTR. Thus, the alternative splicing of noncoding exons would lead to the same protein sequence [4]. Different amino acid sites play different roles in determining the specificity of receptors. Once a matched ligand molecule reaches a receptor, the cell can react to this signal. Any OR gene can produce a receptor protein, which helps animals to distinguish many different compounds [5, 6]. According to differences in their amino acid sequences, receptor proteins are usually classified into Class I and Class II proteins [7–9]. Although the functional difference between these two classes is still unclear, the former tends to bind water-soluble odor molecules, while the latter tends to bind hydrophobic odor molecules [6]. The majority of ORs in fish are Class I receptors [10], whereas the majority of amphibians and mammals harbor Class II receptors [8].

Previous studies showed that the OR repertoire varies greatly among different species [11], which is mostly due to the different ecological niches for each species [12, 13]. On the one hand, the number of OR genes varies among mammals [13]. For example, elephants have the largest OR repertoire encoded in enlarged gene clusters among mammals, and cetaceans have the small number [14, 15]. On the other hand, different mammals have similar numbers but different repertoires [16]. In chimpanzees and humans, they have a similar number of intact OR genes, but approximately 25% of these intact genes are not homologous [17]. Accordingly, sensory functions, such as taste and olfaction, are generally reduced in marine mammals because their sensory systems have evolved to adapt to underwater life through an emphasis on light and sound sensing [18]. Actually, all cetaceans underwent a significant loss of olfactory-functional ORs during the land to water transition [19]. Another interesting example is that platypuses are a semiaquatic and egg-laying mammal with relatively few intact OR genes (approximately 350) [16], and the gene number is probably low because platypuses have electroreceptors that can sense subtle electronic changes.

In addition to the variation of OR gene numbers among mammal species, OR genes have experienced frequent gains and losses during evolution [11, 14, 16]. Niimura et al. found that the gains and losses of OR genes have occurred in an order-specific pattern [11, 16]. Therefore, the OR gene family is considered an extreme example of gene family expansion and contraction [20]. New OR genes were generated through gene duplication events, while some genes were lost through pseudogenization. For instance, the human genome encodes approximately 400 OR genes. Intriguingly, this genome also contains more than 400 OR pseudogenes [8, 14]. In summary, members of the OR gene family have changed greatly during the evolution of mammals. Some ancestor OR genes may have produced large numbers of new genes in different lineages, while others may have been lost shortly after their creation. Of course, some genes remain evolutionarily stable because of a lack of gene duplication or loss [14].

Mammals have experienced several independent evolutionary events from terrestrial environments back to aquatic environments. More than 120 extant species of marine mammals have been identified, and they belong to three different mammal lineages: Pinnipedia (such as seals, sea lions and walruses), Cetacea (such as whales, dolphins and porpoises) and Sirenia (such as manatees and dugongs). Because of their independent involvement in different periods and many shared features, marine mammals are generally regarded as a typical example of convergent evolution [21, 22]. Hughes et al. showed that OR gains and losses are correlated with environmental adaptations [13]; thus, it is interesting to study the repertoire change of OR genes among mammalian lineages, especially between terrestrial and aquatic mammals. Accordingly, OR gains and losses occurred frequently during evolution, and the number of OR members varies greatly among different species. However, it is still unclear whether the extent of gene gains/losses varies between marine and terrestrial mammals and what factors underlie these variations. Although we cannot predict the evolutionary fate of genes, we can trace the evolutionary trajectory of genes by comparing them among species. Therefore, we compared the gene number and orthologous gene groups (OGGs) of marine and terrestrial mammals in this study and found that the convergent degeneration of OR genes occurred among independent marine mammalian lineages. The results could help us to understand the gene gains and losses of different mammalian evolutionary lineages during the process of re-adaptation to aquatic environments.

## Results

### OR gene numbers of marine mammals are significantly lower than those of terrestrial mammals

**Fig. 1:**
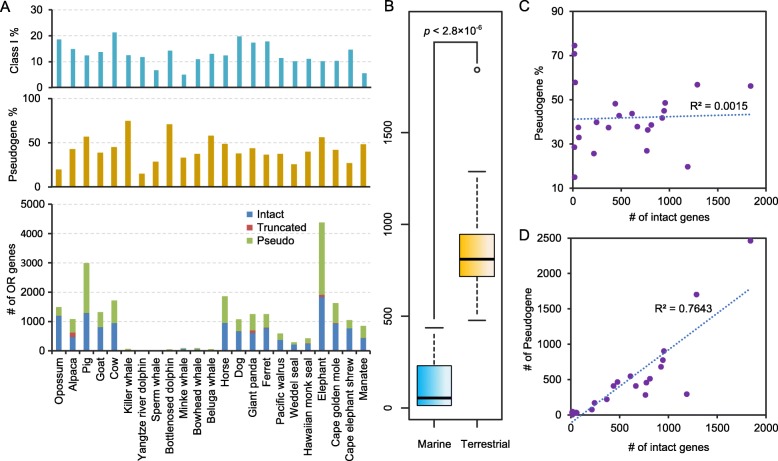
Comparison of the number of OR genes encoded in mammalian genomes. **a** Class I gene proportion refers to the number of intact Class I genes divided by the total number of all intact OR genes in the species. Pseudogene proportion refers to the number of pseudogenes divided by the total number of OR genes in the species. The OR gene information of opossum was obtained from [16]. **b** Number of intact OR genes was compared between marine mammals and terrestrial mammals using the Mann-Whitney U test (*p* value < 2.8 × 10^− 6^). **c** Significant correlations were not observed between the proportion of OR pseudogenes in each genome and the number of intact OR genes (Pearson's correlation coefficient *r* = 0.039; *p* value = 0.86). **d** Absolute number of OR pseudogenes was positively correlated with the number of intact genes (*r* = 0.874; *p* value = 4.99 × 10^− 8^)

We identified a total of 12,711 intact OR genes from the full genome data of eutherian mammals (including 11 marine mammals and 11 terrestrial mammals) and the outgroup opossum genome based on protein sequence similarity and homologous relationships. Figure 1a and Additional file 2: Table S1 show detailed information on these results. We found that the OR gene numbers in marine mammals were significantly lower than those in closely related terrestrial mammals (Fig. 1b, Mann-Whitney U test, *p* value *=* 2.84 × 10^− 6^), which is consistent with previous reports. Furthermore, the number of OR genes in cetacean species (14~61) was very different from the number of OR genes in terrestrial mammals (484~1680). In Pinnipedia and Sirenia, the number of OR genes was relatively high, with 217~369 OR genes in Pinnipedia and 438 OR genes in manatee, although the number was still significantly lower than the OR gene number of terrestrial mammals.

The number of OR genes varied greatly among different species (Fig. 1a). Among the 23 species we analyzed, African elephants had the largest number of intact OR genes (1841) and pseudogenes (2462), and the number of OR genes and pseudogenes was more than twice higher than that of close relatives. This result is basically consistent with previous studies [14]. Similarly, the proportion of OR pseudogenes also varied greatly among different species (Fig. 1a, Additional file 2: Table S1). Killer whales had the highest proportion of OR pseudogenes (75%), and Yangtze River dolphins had the lowest proportion of OR pseudogenes (15%). As shown in Fig. 1c, significant correlations were not observed between the proportion of OR pseudogenes in each genome and the number of intact OR genes (Pearson's correlation coefficient *r* = 0.039; *p* value = 0.86). Therefore, the proportion of OR pseudogenes cannot be used to predict the number of intact OR genes for a particular genome. In contrast, the absolute number of OR pseudogenes was positively correlated with the number of intact genes (Fig. 1d, *r* = 0.874; *p* = 4.99 × 10^− 8^).

### The OGG numbers of marine mammals are significantly lower than those of terrestrial mammals

In this study, we obtained a total of 1111 OGGs, of which 281 OGGs contained only one OR sequence; thus, in subsequent analyses, we only used the 830 OGGs containing at least two sequences. Based on the principle of similarity to intact OR gene sequences, we also classified all truncated genes and pseudogenes into the 830 OGGs (see Methods for details). According to the definition of orthologs, in an OGG, all genes are derived from the most recent common ancestor (MRCA). Therefore, we speculate that there are approximately 830 intact OR genes from the MRCAs of the studied marine mammals and their closely related terrestrial mammals. These genes varied among different species due to gene gains and losses.

**Fig. 2:**
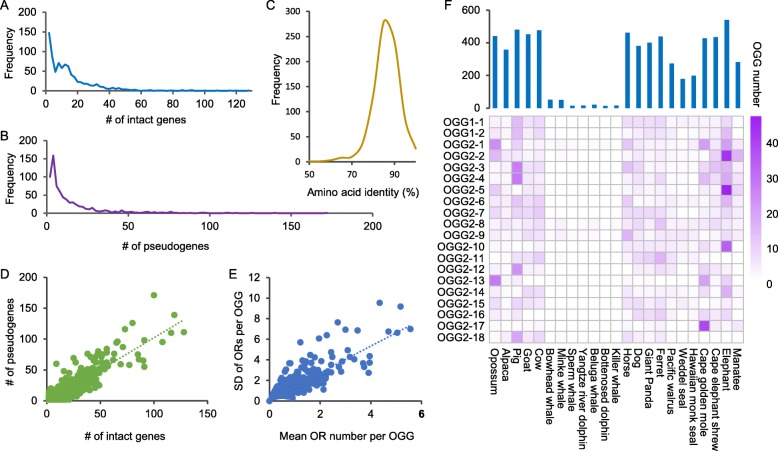
Comparison of the gene number and sequence similarity in each OGG. **a** and **b** Distribution of the number of intact OR genes and pseudogenes among OGGs. **c** Distribution of average amino acid similarity among genes in each OGG. **d** Number of intact OR genes in different OGGs was positively correlated with the number of pseudogenes in the corresponding OGGs (*r* = 0.886, *p* value < 2.2 × 10^− 16^). **e** Difference in the mean number of OR genes per OGG among different species was positively correlated with the average OGG size (*r* = 0.860, *p* value < 2.2 × 10^− 16^). **f** Distribution of the OGG number among different species (upper panel); and gene numbers of the 20 largest OGGs (lower panel)

As shown in Fig. 2a and b, most OGGs contained a small number of OR genes and pseudogenes. Among the 830 OGGs, the average and median numbers of intact genes per OGG were 15.0 and 11, respectively, and for pseudogenes, the mean and median numbers were 14.9 and 7, respectively. We also calculated the average sequence similarity among different genes in the same OGG, and the majority showed 80%~ 90% similarity (Fig. 2c). The similarity was relatively low in large OGGs and relatively high in small OGGs. For all OGGs, the number of intact OR genes was positively correlated with the number of pseudogenes (Fig. 2d, *r* = 0.886, *p* value < 2.2 × 10^− 16^). That is, OGGs with more intact genes possessed more pseudogenes. In the same OGG, although the OR gene sequences were relatively conserved, the OR gene number was relatively highly variable among species. To investigate the differences in the OR gene numbers of different species, we compared the relationship between the standard deviation and total number of OR genes in each OGG and found that they were significantly positively correlated (Fig. 2e, *p* value < 2.2 × 10^− 16^). In other words, smaller-sized OGGs were correlated with smaller differences among species, which indicates that large-scale OGGs generally tend to be subject to an extreme form of birth-and-death evolution [20, 23]. This pattern is more common in gene family evolution, and this phenomenon is mainly caused by tandem gene duplication [24].

Simionato et al. [25] reported that gene family size does not generally reflect the evolutionary diversity of gene families, such as the tyrosine kinase family and the basic helix-loop-helix family [25–27]. Therefore, we tried to explore the difference in the OR gene numbers between marine and terrestrial mammals resulting from gene-specific duplications or increased numbers of gene gains and losses. As shown in Fig. 2f, we compared the OGG numbers among 23 species. The number of OGGs also varied greatly among different species and ranged from 13 to 541. Significant differences were observed in the number of OGGs between marine and terrestrial mammals (Mann-Whitney U test, *p* value = 5.53 × 10^− 5^). Then, we selected the 20 largest OGGs and found that there were large numbers of species-specific duplications in these OGGs. For instance, more than 30 members were included in OGG2–2, OGG2–5, and OGG2–10 in elephant and OGG2–17 in cape golden mole.

### OR genes experienced gains and losses under weaker evolutionary constraints

**Fig. 3:**
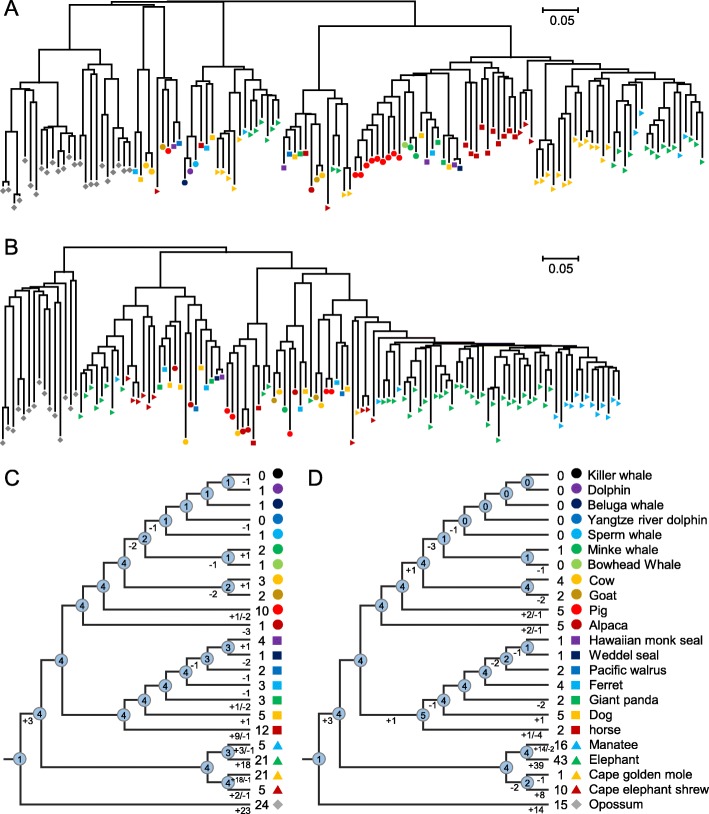
Expansion of the OR gene in mammals. **a** and **b** NJ phylogenetic trees constructed from all intact OR genes in OGG2–1 and OGG2–2, respectively. **c** and **d** Number of gene amplifications and losses in the OGGs of (**a**) and (**b**) in different branches and nodes, respectively

The sizes of some OGGs are very large, indicating that some ancestor OR genes experienced large numbers of duplications in certain mammals (as shown in Fig. 3a, b). OGG2–1 contained the largest number of intact OR genes (128), particularly in opossum, cape golden mole and elephant (> 20 intact OR genes), and OGG2–2 was the second largest OGG and contained 119 intact OR genes, with the most OR genes in elephant (43). The phylogenetic analysis indicated that these large OGGs originated from a large number of independent gene gains and losses among different species (Fig. 3c, d). Comparing the distribution of marine and terrestrial mammals in different OGGs, we found that the loss of the ancestral OR gene occurred in different marine lineages. For example, in OGG2–1, cetaceans lost two of their four ancestral genes, and only one of the remaining two ancestral genes was retained by different cetacean species. One gene was also lost in the ancestral state in Pinnipedia (Fig. 3c). For OGG2–2 in the cetacean lineage, only the minke whale retained an intact OR gene, while all genes were lost in the other species; moreover, two of these OR genes were lost in the ancestors of Pinnipedia (Fig. 3d). Additionally, OGG2–5 contained the largest number of pseudogenes (171).

We calculated the species-specific gain and loss rates for each OGG in each species and considered the phylogenetic relationships among species, which represent the extent of branch-specific gene gains or losses in the 23 mammals. The results indicate that specific gene gains were frequent in elephant and opossum, and genes were often lost in marine lineages, especially in cetaceans [13].

**Fig. 4:**
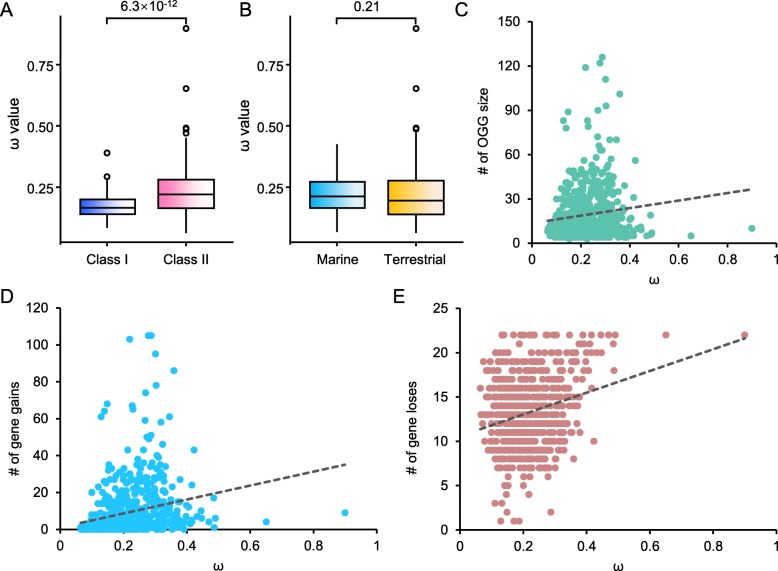
Comparative analysis of OR gene selective pressure. **a** Boxplots of the comparison between Class I (blue) and Class II (pink) OGGs for estimated ω values. **b** Boxplots of the comparison between the estimated ω values of OGGs containing marine mammals and excluding terrestrial mammals (Mann-Whitney U test used to test the difference). **c** Number of intact genes in each OGG is positively correlated with the estimated ω value of the respective OGG, and the dashed line indicates the regression line. **d** Estimated ω value for an OGG was positively correlated with the gene amplification number in the respective OGG. **e** Estimated ω value for an OGG was positively correlated with gene loss number in the respective OGG. The correlation coefficients (*r*) of (**c**), (**d**) and (**e**) are 0.129, 0.221 and 0.253, and the *p* values are 1.24 × 10^− 3^, 2.51 × 10^− 8^ and 1.56 × 10^− 10^, respectively

Then, we used the maximum likelihood method in PAML 4.9 to estimate the nonsynonymous/synonymous replacement rate (ω value) of each OGG. This value reflects the extent of purifying selection. In a comparison of the Class I and Class II genes, the former was found to be significantly smaller than the latter (Fig. 4a, *p* value < 6.3 × 10^− 12^), indicating that the Class II genes are more dynamic than the Class I genes during evolution. As shown in Fig. 4b, no significant difference was found in a comparison between the estimated ω values of OGGs containing marine mammal genes and marine mammal-free OGGs. The estimated ω values of OGGs containing the three marine lineages were indistinguishable from the other OGGs or all OGGs (Additional file 1: Figure S1). This finding may be due to the small number of marine OR genes, which were easily overwhelmed by the background branching noise of the OGGs. The estimated ω value was also positively related to the number of intact OR genes in the OGGs (*r* = 0.129, *p* value *=* 1.24 × 10^− 3^) (Fig. 4c). The estimated ω value of each OGG was positively correlated with the number of gene gains in the OGG (*r* = 0.221, *p* value *=* 2.51 × 10^− 8^) (Fig. 4d). Moreover, the estimated ω value of each OGG was also positively correlated with the number of gene losses in the OGG (*r* = 0.253, *p* value *=* 1.56 × 10^− 10^) (Fig. 4e). These analyses suggested that OGGs having undergone more gene gains or losses are often under weaker evolutionary constraints.

### OR genes in marine mammals are not evolutionarily conserved

**Fig. 5:**
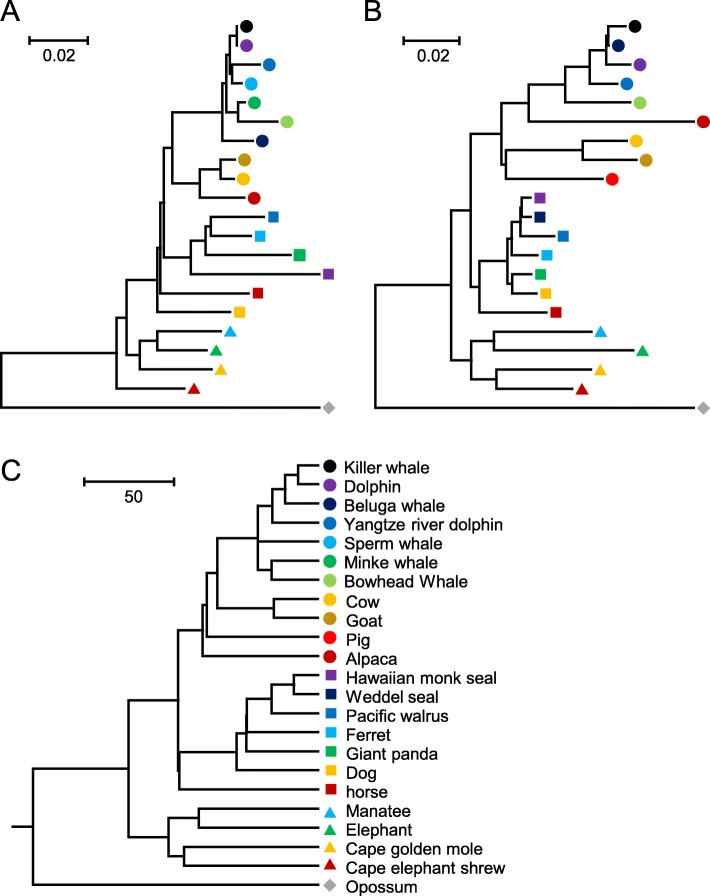
NJ phylogenetic trees of single-copy OR genes in marine mammals and their related terrestrial mammals. **a** and **b** NJ phylogenetic trees for OGG1–22 and OGG1–23, respectively. **c** Species tree used in this study

Among the 830 OGGs, we did not find any OGG containing OR genes from all 23 mammals, indicating that the OR genes showed evolutionary diversity between marine and terrestrial mammals, and this phenomenon may be related to differences in their environments. Moreover, we also failed to find OGGs containing the genes of all species from the three marine lineages. We found two OGGs (lost in one or more species) containing a single copy of each species, i.e., OGG1–22 and OGG1–23. As shown in Fig. 5a, OGG1–22 was lost in the Weddell seal and pig but presented as a single copy in other species, and no pseudogenes were found in this OGG. However, the phylogenetic analysis of this OGG did not exhibit a topology similar to that of the species tree, indicating that this gene was not very evolutionarily conserved. As shown in Fig. 5b, OGG1–23 did not contain minke whale and sperm whale genes and presented as a single copy in the other species, with two opossum pseudogenes. The phylogenetic analysis revealed that the members of this OGG exhibited a topology similar to that of the species tree (Fig. 5c), indicating that genes in this OGG were truly orthologous among species, and no gene gain and loss events occurred during evolution. In other OGGs, different degrees of gene gains and losses occurred. No OR orthologous genes, including the above two OGGs, were found in all marine mammals, indicating that the methods of OR degradation or loss in different lineages are not the same.

### Marine mammals show a lower rate of gene gains but a higher rate of gene losses than terrestrial mammals

**Fig. 6:**
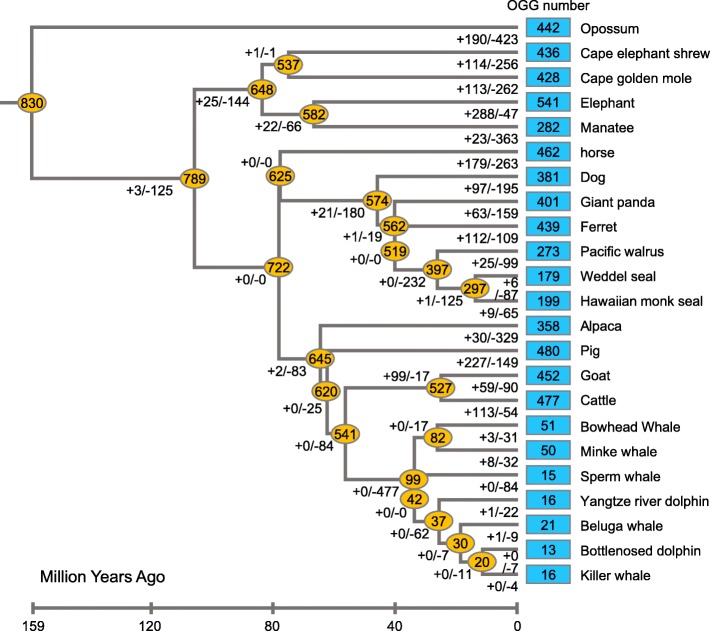
Changes in OGGs during the evolution of marine mammals and their related terrestrial mammals. The numbers of OGGs with intact OR genes for different species are shown in blue boxes. The numbers of OGGs with intact OR genes on the ancestor node are shown in yellow ellipses. Additionally, the numbers of OGGs that expanded and contracted (or were lost) are shown on each branch. For instance, 190 of 830 OGGs were gained by expansion in opossum, but 423 OGGs were contracted or lost (35 contractions and 388 losses), resulting in 442 intact OR genes in opossum. Notably, because the number of OGG contractions (or losses) includes both gene size reduction and total loss, the number of MRCAs minus the number of OGG contractions (or losses) on the branch does not necessarily equal the number of OGGs on the next node. The divergence time of each node was obtained from the TimeTree (http://www.timetree.org/) database

During the evolution of marine mammals and their closely related terrestrial mammals, we estimated the OGG gain and loss rates of 830 OR genes on each branch. Consistent with previous studies, large numbers of gains and losses occurred in different branches [14, 16] (Fig. 6). Thus, although two species may have similar numbers of OGGs or genes, they may have very different OR repertoires. For example, both bowhead whale and minke whale had approximately 50 OGGs, whereas only 19 OGGs were common (< 40%). The cape golden mole and cape elephant shrew presented 327 shared OGGs, which accounted for approximately 76% of all OGGs. Additionally, each of the 23 mammals clearly lost hundreds of intact OR genes present in MRCAs, although the number of genes lost in the three marine lineages was greater than that lost in the terrestrial lineages (Fig. 6, Mann-Whitney U test, *p* value = 5.56 × 10^− 5^). There were 99 intact OR genes in the cetacean MRCA, approximately 88% of which were lost; there were 397 intact OR genes in the Carnivora MRCA, approximately 52% of which were lost; and there were 282 intact OR genes in the MRCA of Pinnipedia, approximately 66% of which were lost.

**Fig. 7:**
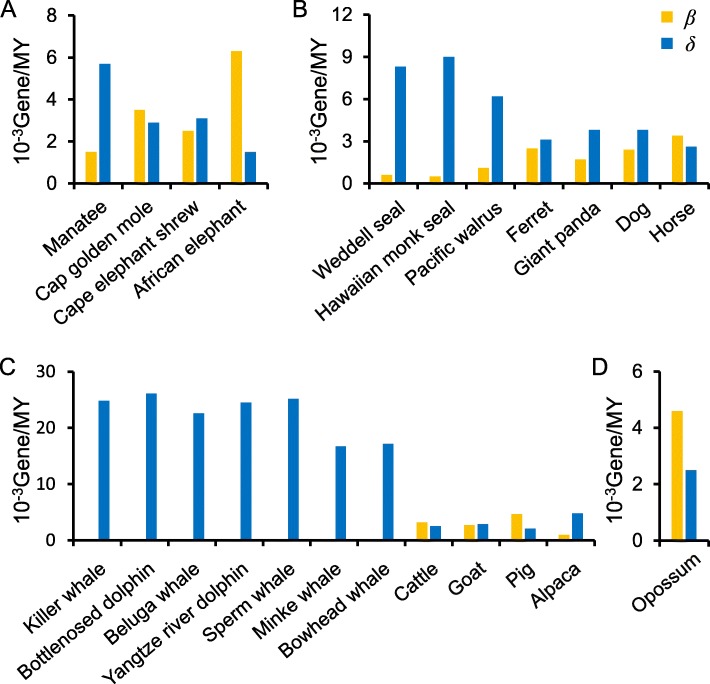
Gain and loss rates of OR genes in marine mammals and related terrestrial mammals. β and δ represent the gain and loss rates of OR genes (gene per MYs)

We also estimated the gain (*β*) and loss (*δ*) rates of the OR genes in each species. *β* and *δ* were defined as the number of gene gains or losses per million years (MYs), respectively, and it was assumed that these two indices were constant on each branch. We calculated the *β* and *δ* of each species by the calculation method of Niimura et al. [14] (Fig. 7). The results show that *β* was largest in African elephants, which is consistent with the results of Niimura et al. [14], while almost no gene gains occurred in the cetacean branches (*β* was between 0 and 0.0002). The *β* values in marine mammals were significantly lower than those in terrestrial mammals (Mann-Whitney U test, *p* value = 8.86 × 10^− 5^), and the *δ* values in marine mammals were significantly higher than those in terrestrial mammals (Mann-Whitney U test, *p* value = 5.46 × 10^− 5^). During the evolution of marine mammals and their related species, the average *β* and *δ* were 0.0016 and 0.0088 (gene per MYs), respectively (Fig. 7). The former was consistent with the average gene family size change rate (turnover, including gain and loss) previously reported in mammalian genes, i.e., 0.0016 per gene per MYs [28]; however, the latter was much larger. To compare the differences between marine and terrestrial mammals, we compared the average *β* and *δ* in both types of mammals. The mean *β* and *δ* in terrestrial mammals were 0.0032 and 0.0030 (per million per gene per year, Fig. 7), respectively, which were both higher than 0.0016, while the average *β* and *δ* in marine mammals were 0.0004 and 0.0169 (gene per MYs), respectively, where the gain rate was significantly lower than the average, and the loss rate was much higher than the average (approximately 10 times).

## Discussion

Previous studies suggest that animals living in different ecological niches require different OR genes [12, 13]. In this study, we identified more than 10,000 OR genes from 23 eutherian mammals to compare the OR repertoire differences among independent lineages and between terrestrial and marine mammals. As early as the end of the last century, studies have found that the number of OR genes in terrestrial quadrupeds is very different from that in marine fishes [29, 30], and these genes generally differentiated after the split between fish and tetrapods [31]. Historically, genomic data on marine mammals were very limited compared to those on terrestrial mammals; however, with the gradual completion of large-scale genome sequencing, techniques for screening intact OR genes and pseudogenes in the genomes of marine mammals and their closely related terrestrial mammals have been developed. Moreover, the changes in OR genes in marine mammals in lineages of independent origin during the process of re-adaptation to aquatic environments from terrestrial environments represent an interesting research topic.

Our analysis shows that cetaceans lost a large amount of OR genes when they became fully adapted to the water environment because they are largely independent of the terrestrial environment throughout their life cycle (Fig. 6). Pinnipedia and Sirenea aquatic mammals still retain large numbers of OR genes because they have a certain dependence on terrestrial or shallow water environments in terms of reproduction or other important functions (Fig. 1a). However, the number of OR genes in marine mammals is significantly lower than that in their related terrestrial mammals. These results show that the highly dynamic evolutionary history of the OR gene family is independent of changes in genomic organization and instead is more likely influenced by special ecological niches [13, 32]. In fact, olfactory bulbs and their related nervous systems in terrestrial animals play an important role in recognizing airborne odors, whereas these systems in marine organisms, such as whales, are reduced or even lost. In modern cetaceans, olfactory sensitivity has been gradually lost or greatly degraded. Similarly, birds are less susceptible to odors than most mammals; therefore, they have fewer intact OR genes than mammals [33]. Higher primates usually have fewer OR genes, which may be related to the fact that primates rely more on sight than smell, although the mechanism for this difference in OR gene number remains unknown [34].

All members of the mammalian OR families consist of functional and nonfunctional genes. Many of these genes that share the same or similar functions may occur in the MRCAs of certain species and may immediately generate new ORs through duplication. The standard deviations of OR genes in each OGG are significantly positively correlated with the total number of each OGG (Fig. 2e), which may indicate that large-scale OGGs have higher gene turnover than small-scale OGGs [24]. OR gene repertoire sizes vary significantly among mammals due to lineage-specific expansions in a few large OGGs, and such expansions may be correlated with their special ecological adaptions. In addition, the fixation or loss of these new genes may be a characteristic of the niches of certain species [13]. Our study indicated that the MRCAs of selected mammalian species have at least 830 intact OR genes and the numbers vary greatly among different branches (Fig. 6, ranging from 13 to 541). These differences are due to species-specific gene gain or loss events among different species. The number of OGGs in terrestrial mammals is significantly higher than that in marine mammals (Fig. 2f), indicating that the loss of OR genes began in the early evolution of aquatic mammals. The number of genes in different OGGs also varies greatly, which is the result of the combination of specific gene gain and loss events in different species. In a comparison of terrestrial and marine mammals, we found that even in large OGGs, the number of marine lineages is small (Fig. 3). Mammals have hundreds of OR genes, each of which has the ability to bind one or more odor molecules [6]. The convergent loss of olfactory genes in marine mammals is most likely due to the unique olfactory system of aquatic mammals, which subjects most OR genes to relaxed or no selective constraints. That is, in some special cases, the loss of limited pleiotropic genes or monofunctional genes may not lead to harmful results and may therefore be allowed during evolution [35].

We found no OGGs that contained all 23 mammals (Fig. 5), and we also did not find any OGGs that contained genes from all three marine species branches, indicating that the convergent degradation of OR genes in different lineages occurred in different ways. We found that marine mammals have a lower number of ORs than their closely related terrestrial lineages, with the lowest number of ORs in the cetacean lineage, which is closely related to olfactory sensory degeneration. OGGs that have undergone more gains or losses are often under weaker evolutionary constraints (Fig. 4). Among the tested species, we did not find one-to-one orthologous genes, indicating that OR genes are not conserved among marine mammals. The average gain and loss rates of OR genes in terrestrial mammals were higher than those of the mammalian gene family, while the average OR gene gain and loss rates of marine mammals were significantly higher and much higher than those of the mammalian gene family, respectively (Fig. 7). Purifying selection is often the primary force that influences gene evolution. If this purifying selection of ORs in some species is relaxed, then the corresponding OR genes may become pseudogenes.

Terrestrial mammals occupy many different niches that may be related to large numbers of OR gene duplications. In aquatic mammals, the relatively small number of OR genes indicates that there is selective pressure in the aquatic environment that relaxes the olfactory mechanism. We also observed some duplicated OR genes in aquatic mammalian branches (e.g., manatee), which were proportionally high compared to that of cetaceans, suggesting that some of the ORs remain functional in the aquatic environment. Even for Class I OR genes (usually bound to water-soluble odor molecules), some terrestrial adaptive species (such as dogs) exhibit high gene gain rates relative to aquatic mammals. This finding may imply that Class I OR genes performed different functions in mammals relative to fish or played less important roles for marine mammals [12], which suggests that this type of gene has gained new functions or collaborative optimization during adaptations to a non-aquatic environment [36].

Overall, the OR genes of marine mammals and terrestrial mammals are significantly different, and most genes that became differentiated in the terrestrial environment gradually degenerate in the water environment due to the loss of functional constraints and the requirement of these genes for survival. Among the 830 OGGs, we failed to find any OGGs that contained OR genes from all 23 mammals or all marine lineages. Two OGGs contained a single copy of majority species, namely, OGG1–22 and OGG1–23, although only the latter exhibited a topology similar to that of the species tree (Fig. 5c), which indicated that OR degradation or loss of three marine mammal lineages is also not conserved. In fact, studies have shown that not only ORs but also taste receptors, vomeronasal receptors, and other receptors are found in terrestrial quadrupeds and marine fish [15, 37, 38]. Therefore, further studies on other sensory receptors, such as taste or hearing receptors, will help us to further understand the molecular mechanisms of information transmission and reception underwater. Our study provides insights into the adaptive evolution of senses in mammals in different niches and useful information on the molecular mechanisms of olfactory diversity in species, especially in marine mammals.

## Conclusions

In summary, the OR genes as well as the taste receptors, vomeronasal receptors, and other receptors of marine mammals and terrestrial mammals are significantly different [2, 38–42], and most genes that became differentiated in the terrestrial environment gradually degenerate in the water environment due to the loss of functional constraints and the requirement of these genes for survival. In this study, we identified more than 10,000 members of the OR family in 23 mammals and classified them into 830 OGGs. The results showed that marine mammals have experienced a large number of OR genes compared with their related terrestrial lineages, which may result from weaker purifying selection. The average gain and loss rates of OR genes in terrestrial mammals were higher than those of mammalian gene families, while the average gain and loss rates of OR genes in marine mammals were significantly lower and much higher than those of the mammalian gene families, respectively. Due to their independent degeneration, the OR genes present in each lineage are not well conserved among marine mammals. Our study may provide a basis for future research on the olfactory receptor function in mammals from the perspective of evolutionary trajectories. Of course, further studies on other sensory receptors, such as taste or hearing receptors, would also provide important insights into the molecular mechanisms of information transmission and reception underwater.

## Methods

### Sequence identification, alignment and evolutionary analysis

The mammalian genome data used in this study were downloaded from the NCBI database. We selected 11 marine mammals (Cetacea, Carnivora, and Sirenia) from 3 independent origins; specifically, these mammals were killer whales, sperm whales, minke whales, bowhead whales, beluga whales, bottlenose dolphins, Yangtze River dolphins, Weddell seals, Hawaiian monk seals, Pacific walruses and manatees, respectively. We also selected 11 terrestrial mammals related with above three marine lineages, respectively. Among them, four species are from Artiodactyla (cattle, pigs, goats and alpacas), three from Carnivora (ferrets, giant pandas and dogs), and three from Afrotheria (golden moles, elephant shrews and African elephants). We also include horse from Perissodactyla, which has a close relationship with Carnivora [43]. Additionally, we used opossum as an outgroup in this study. As Hayden et al. (2010) mentioned, significant differences occurred between the number of pseudogenes but did not occur in the functional OR gene repertoire between low and high coverage assemblies [12]. Therefore, the genomes of species with varying quality cannot contribute towards the OR differences between marine mammals and their related terrestrial lineages.

The methods for identifying OR genes from the genome were as described in Niimura (2013) [9]. First, we obtained all the OR genes identified in humans and mice and then only kept each gene that shared less than 50% protein sequence identity to another, so as to capture the diversity of sequences and remove redundant blast hits. Thus, the retained 85 intact OR genes distributed in two classes (I and II) were used as query sequences. tBLASTn searches were conducted against the genomes of the studied species, and the *E* value was set to 1 × 10^− 10^. For the hit regions, we selected the regions with the highest scores and extracted the sequences. Sequences shorter than 250 amino acids in length were discarded. The remaining sequences were extended to both ends, and the longest sequences that started with an ATG start codon, ended with a stop codon (TAA, TAG or TGA) and had no stop codons in the protein-coding region were kept for further study. These sequences were subjected to multiple sequence alignment using the E-INS-i program in MAFFT using the default parameters [44] and then divided into seven transmembrane regions according to Man et al. [45]. If at least one transmembrane region of a sequence had a gap greater than 5 amino acids in length [9], it was discarded. If there were multiple ATG codons at the N-terminus, the start codon position was determined based on the principle that the N-terminus (the length to the first transmembrane region) must be 21 to 34 amino acids in length.

Then, we determined whether the obtained sequences were OR genes by constructing phylogenetic trees. We aligned the sequences from each species using the E-INS-i program in MAFFT with default parameters [44] and constructed phylogenetic trees using the neighbor-joining (NJ) method inbuilt in MEGA 6 based on Poisson distances [46] or the Maximum Likelihood (ML) method implemented in RaxML [47] based on the JTT model with 1000 bootstraps. The following non-OR GPCR genes were used as outgroups: alpha-1A-adrenergic receptor isoform 1 (GenBank protein ID, NP_000671), 5-hydroxytryptamine (serotonin) receptor 6 (NP_000862), galanin receptor 1 (NP_001471), 5-hydroxytryptamine (serotonin) Receptor 1F (NP_000857), histamine receptor H2 (NP_071640), adenosine A2b receptor (NP_000667), beta-1-adrenergic receptor (NP_000675), somatostatin receptor 4 (NP_001043), 5-hydroxytryptamine (serotonin) receptor 1B (NP_000854), Mouse GPCR148 (AY569570–1) and putative GPCR. When the tested gene clustered into an outgroup, we regarded it as a non-OR gene and removed it.

Finally, the OR genes obtained as described above were considered intact OR genes and used as query sequences in tBLASTn searches (*E* value 1 × 10^− 20^) against the corresponding genome. The reason for this was to both find all pseudogenes/truncated genes and to find any intact genes potentially missed by initial genome sweeps for OR sequences using human and mouse genes as a query. For matches, we selected the regions with the highest score. The intact OR genes were removed, and all remaining genes were considered pseudogenes or truncated genes. If the gene met the following two conditions, it was regarded as a truncated gene: 1. there was no stop codon or frameshift mutation in the sequence; and 2. the distance between the end of the sequence and the end of the contig was less than 30 bp.

### Gene classification and orthologous gene group assignment

Due to the large number of OR genes in mammals, we analyzed the OR genes according to two aspects, namely, Class I and Class II classification and OGG assignment. First, we constructed a phylogenetic tree for the OR genes of each species. Generally, the Class I and Class II OR genes formed two different branches, and the Class I ORs were located basal to the Class II ORs in the phylogenetic tree. From these results, we roughly classified the OR genes of each species into Class I and Class II patterns. Then, according to Niimura et al., we used 33 single-source evolutionary branches (one Class I and 32 Class II branches, the latter named A-S, AA-AJ, AT, BB, and BC), and the OR genes were classified into different phylogenetic branches [48, 49] with high bootstrap (90%) support. Thus, we classified all identified OR genes into Class I or Class II.

Then, we used the following method to assign OGGs. We used OrthoMCL [50] to classify all the identified intact OR genes and obtained 747 OGGs containing at least two members. The remaining 491 individual genes were classified by a phylogenetic tree analysis. First, we constructed a phylogenetic tree for all 491 OR gene sequences as well as non-OR GPCR genes and set the non-OR GPCR genes as outgroups. In this phylogenetic tree, if different genes were clustered together and the bootstrap value was greater than 70, they were classified into one OGG, and the rest were considered to be single genes. Thus, we classified 491 individual genes into 364 OGGs. Among the 23 mammals, we assigned 12,711 intact OR genes into 1111 OGGs, with 830 OGGs containing more than 2 ORs and 281 OGGs containing only one OR. The OGGs were named as follows: first, they were named OGG1 or OGG2 if they belonged to Class I or Class II, respectively, and then were assigned numerical values according to the OGG size, where the largest OGG was named OGG1–1, etc.

Finally, we classified all the nonintact genes (including pseudogenes and incomplete genes) into the above 1111 OGGs. Due to the inaccuracy of evolutionary relationships inferred by segment sequences, we directly used the BLAST best matching method for classification. In other words, each nonintact OR gene was subjected to a BLAST search against 12,711 intact OR genes and classified into the OGG where the best sequence was located.

### Gene gain and loss rate estimation

The gain rate (*β*) usually refers to the number of intact genes gained per MYs, and the loss rate (*δ*) refers to the number of intact genes lost per MYs. We calculated the *β* and *δ* of each branch in the phylogeny of 23 mammals. For each branch, *β* and *δ* were assumed to remain constant over time. Suppose that there are *A*_*0*_ genes at the start time *t* = 0. At *t* = *T*, the number of genes becomes *A*_*0*_ + *G* - *L* due to gene gains (*G*) and gene losses (*L*) during this time. The number of gene gains until time *t* is *g(t)*, and the number of losses is *l(t)*. Therefore, *G* = *g(T)* and *L* = *l(T)*. Then the following formula can be obtained [14]:

$$ \frac{dg(t)}{dt}=\left({A}_0+g(t)-l(t)\right)\beta $$

$$ \frac{dl(t)}{dt}=\left({A}_0+g(t)-l(t)\right)\delta $$

From this, we can obtain the following formula:

$$ \beta =\frac{G}{\left(G-L\right)T}\ln \left(1+\frac{G-L}{A_0}\right) $$

$$ \delta =\frac{L}{\left(G-L\right)T}\ln \left(1+\frac{G-L}{A_0}\right) $$

Using the above formula, we can calculate the *β* and *δ* values.

### Selective pressure analysis

We used the likelihood algorithm in PAML 4.9 [51] to estimate the nonsynonymous/synonymous replacement rate ω. Here, we only analyzed OGGs with 3 or more sequences. We constructed these OGG unrooted trees and used the Codeml with one ratio (M0) model and F3 × 4 codon frequency models to calculate the global ω values.

## Supplementary information

**Additional file 1: Figure S1.** Comparison of estimated ω values of OGGs containing three marine lineages with the rest of the OGGs and all OGGs.

**Additional file 2: Table S1.** OR number distribution of 23 mammals. Intact genes indicate coding sequences starting from start codons and ending with stop codons without any interference mutations. Pseudogenes indicate sequences containing nonsense mutations, coding shifts, deletions in conserved regions, or combinatorial features. Truncated genes indicate sequences with partial sequences or located at the end of a contig. Truncated genes can also be pseudogenes. Pseudogene proportion refers to the number of pseudogenes divided by the total number of OR genes in the species.

## Data Availability

All data analyzed during this study are included in this article and its additional files.

## Abbreviations

GPCR: G protein-coupled receptor
MRCA: The most recent common ancestor
OGG: Orthologous gene group
OR: Olfactory receptor
