# Supplementary figures and images for: Convergent degeneration of olfactory receptor gene repertoires in marine mammals
Source: BMC Genomics. 2019 Dec 12;20:977. doi: 10.1186/s12864-019-6290-0 (PMC6916060; doi:10.1186/s12864-019-6290-0)

A

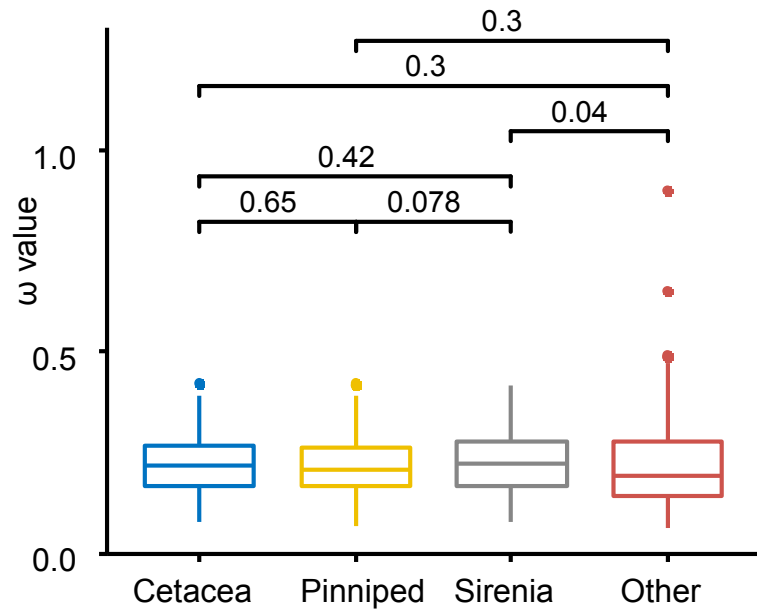

B

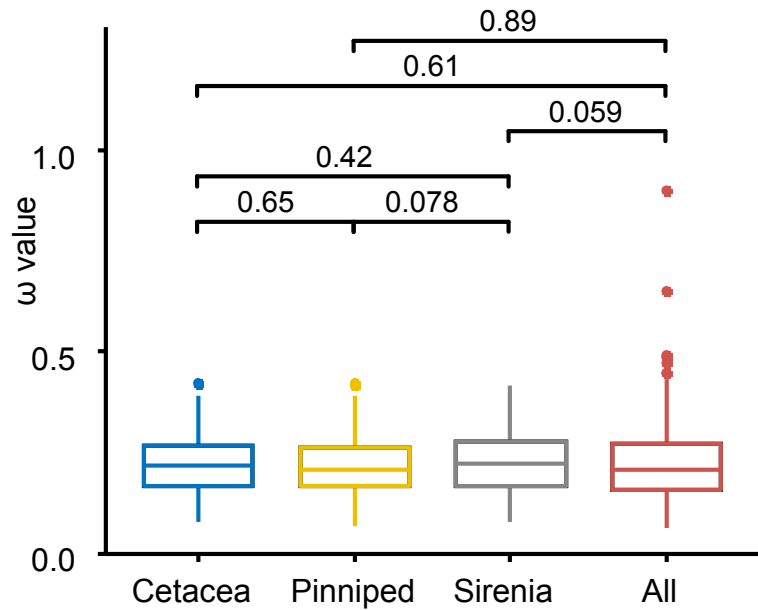

Supplement: Supplementary file 1 — Additional file 1: Figure S1. Comparison of estimated ω values of OGGs containing three marine lineages with the rest of the OGGs and all OGGs. [file 12864_2019_6290_MOESM1_ESM.pdf]
