# Supplementary material for: Convergent degeneration of olfactory receptor gene repertoires in marine mammals
Source: BMC Genomics. 2019 Dec 12;20:977. doi: 10.1186/s12864-019-6290-0 (PMC6916060; doi:10.1186/s12864-019-6290-0)
Supplement: Supplementary file 2 — Additional file 2: Table S1. OR number distribution of 23 mammals. Intact genes indicate coding sequences starting from start codons and ending with stop codons without any interference mutations. Pseudogenes indicate sequences containing nonsense mutations, coding shifts, deletions in conserved regions, or combinatorial features. Truncated genes indicate sequences with partial sequences or located at the end of a contig. Truncated genes can also be pseudogenes. Pseudogene proportion refers to the number of pseudogenes divided by the total number of OR genes in the species. [file 12864_2019_6290_MOESM2_ESM.docx]

**Table S1. OR number distribution of 23 mammals.**

| Order | Species | Intact gene | Truncated gene | Pseudogene | Number | % Pseudogene |
| --- | --- | --- | --- | --- | --- | --- |
| Marsupialia | Opossum | 1188 | 10 | 294 | 1492 | 19.7 |
| Artiodactyla | Alpaca | 478 | 141 | 464 | 1083 | 42.8 |
| Artiodactyla | Pig | 1288 | 2 | 1701 | 2991 | 56.9 |
| Artiodactyla | Goat | 811 | 0 | 511 | 1322 | 38.7 |
| Artiodactyla | Cattle | 943 | 3 | 774 | 1720 | 45 |
| Artiodactyla | Killer whale | 16 | 0 | 47 | 63 | 74.6 |
| Artiodactyla | Yangtze river dolphin | 17 | 0 | 3 | 20 | 15 |
| Artiodactyla | Sperm whale | 15 | 0 | 6 | 21 | 28.6 |
| Artiodactyla | Bottenosed dolphin | 14 | 0 | 34 | 48 | 70.8 |
| Artiodactyla | Minke whale | 61 | 0 | 30 | 91 | 33 |
| Artiodactyla | Bowhead whale | 55 | 0 | 33 | 88 | 37.5 |
| Artiodactyla | Beluga whale | 23 | 1 | 33 | 57 | 57.9 |
| Perissodactyla | Horse | 954 | 1 | 903 | 1858 | 48.6 |
| Carnivora | Dog | 666 | 3 | 408 | 1077 | 37.9 |
| Carnivora | Giant panda | 611 | 92 | 547 | 1250 | 43.8 |
| Carnivora | Ferret | 774 | 23 | 455 | 1252 | 36.3 |
| Carnivora | Pacific walrus | 369 | 0 | 221 | 590 | 37.5 |
| Carnivora | Weddell seal | 217 | 0 | 75 | 292 | 25.7 |
| Carnivora | Hawaiian monk seal | 244 | 14 | 171 | 429 | 39.9 |
| Proboscidea | African elephant | 1840 | 73 | 2462 | 4375 | 56.3 |
| Afrosoricida | Cape golden mole | 924 | 22 | 679 | 1625 | 41.8 |
| Macroscelidea | Cape elephant shrew | 765 | 1 | 282 | 1048 | 26.9 |
| Sirenia | Manatee | 438 | 1 | 409 | 848 | 48.2 |
